# Signal intensity ratio of draining vein on silent MR angiography as an indicator of high-flow arteriovenous shunt in brain arteriovenous malformation

**DOI:** 10.1007/s00330-021-08170-8

**Published:** 2021-07-15

**Authors:** Chun-Xue Wu, Zhen-Xiang Zang, Tao Hong, Meng-Qi Dong, Yi Shan, Zhi-Lian Zhao, Cheng-Bei Hou, Jie Lu

**Affiliations:** 1 Department of Radiology and Nuclear Medicine, Xuanwu Hospital, Capital Medical University, 45# Changchun Street, Xicheng District, Beijing, China; 2 Beijing Key Laboratory of Magnetic Resonance Imaging and Brain Informatics, Beijing, China; 3 Department of Neurosurgery, Xuanwu Hospital, Capital Medical University, Beijing, China; 4 Center for Evidence-Based Medicine, Xuanwu Hospital, Capital Medical University, Beijing, China

**Keywords:** Magnetic resonance imaging, Magnetic resonance angiography, Arteriovenous malformations, Brain

## Abstract

**Objectives:**

To evaluate whether the signal intensity ratio (rSI) of the draining vein on silent MR angiography is correlated with arteriovenous (A–V) transit time on digital subtraction angiography (DSA), thereby identifying high-flow A–V shunt in brain arteriovenous malformation (BAVM), and to analyze whether the rSI and the characteristic of draining veins on silent MRA are associated with hemorrhage presentation.

**Methods:**

Eighty-one draining veins of 46 participants with BAVM (mean age 33.2 ± 16.9 years) who underwent silent MRA and DSA were evaluated retrospectively. The correlation between the rSI of the draining vein on silent MRA and A–V transit time on DSA was examined. The AUC-ROC was obtained to evaluate the performance of the rSI in determining the presence of high-flow A–V shunt. The characteristics of draining veins with the maximum rSI (rSImax) were further compared between the hemorrhagic and non-hemorrhagic untreated BAVM.

**Results:**

The rSI of each draining vein on silent MRA was significantly correlated with A–V transit time from DSA (*r =* −0.81, *p* < .001). The AUC-ROC was 0.89 for using the rSI to determine the presence of high-flow A–V shunt. A cut-off rSI value of 1.09 yielded a sensitivity of 82.4% and a specificity of 82.8%. The draining vein with rSImax and no ectasia was significantly more observed in the hemorrhagic group (*p* = 0.045).

**Conclusions:**

The rSI of the draining vein on silent MRA is significantly correlated with A–V transit time on DSA, and it can be used as an indicator of high-flow A–V shunt in BAVM.

**Key Points:**

• *The signal intensity ratio (rSI) of the draining vein on silent MRA significantly correlated with arteriovenous (A–V) transit time of brain arteriovenous malformation (BAVM) on digital subtraction angiography (DSA).*

• *The area under the receiver operating characteristic curve (AUC) was 0.89 for using the rSI of draining veins to determine high-flow A–V shunt.*

• *Draining veins with maximum rSI and no ectasia were significantly more observed in the hemorrhagic group of BAVM (p = 0.045).*

**Supplementary Information:**

The online version contains supplementary material available at 10.1007/s00330-021-08170-8.

## Introduction

Brain arteriovenous malformation (BAVM) is a cerebrovascular disease in which arteries connect directly with veins without intervening capillaries [1, 2]. The main treatment modalities for BAVM include surgery, radiosurgery, and endovascular treatment [2, 3]. Previous studies have indicated that BAVM with high-flow arteriovenous (A–V) shunt are associated with increased perioperative hemorrhagic events and higher risk of incomplete obliteration [4–7]. Therefore, identification and targeted embolization of high-flow A–V shunt in BAVM are crucial in endovascular management before radiosurgery or surgical resection.

Digital subtraction angiography (DSA) is the gold standard for evaluation of high-flow A–V shunt owing to its high spatial and temporal resolution. However, DSA is an invasive examination with unelectable risks of procedure-related complications and therefore not applicable for serial follow-up examinations. Non-invasive imaging methods such as time-resolved contrast-enhanced MRA and unenhanced time-resolved phase-contrast MRA can provide hemodynamic information, yet they require injection of contrast agent or have limited temporal resolution [8–10].

Silent MR angiography is a new imaging approach based on arterial spin labeling (ASL) and zero time echo (ZTE) techniques and does not require a contrast agent [11]. Several studies have reported that silent MRA achieved better performance in depicting the nidus and draining veins of BAVM compared to time-of-flight MR angiography (TOF MRA) [12–14]. In these studies, a portion of the draining veins of BAVM still could not be observed on silent MRA. Meanwhile, in previous studies on cerebrovascular diseases, signal loss on silent MRA was also found in vessels with slow flow [15, 16]. In ASL, the signal decreases with the transit time from the labeling plane due to T1 recovery, and this might limit slow inflow imaging on silent MRA [16]. Based on these findings, we infer that the SI of draining veins on silent MRA might potentially be associated with blood flow velocity and thereby can be used to identify high-flow A–V shunt.

In addition, some studies indicated that a BAVM hemorrhage is associated with unbalanced inflow and outflow [8, 17]. That is, inflow is rapid, whereas outflow remains unchanged or is reduced. Therefore, we assume that the relative signal intensity and the characteristic of draining veins on silent MRA can reflect the inflow and outflow characteristics respectively. Comparing their characteristics in hemorrhagic and non-hemorrhagic groups of BAVM would be helpful to explore their potential role in BAVM hemorrhage.

Therefore, our hypotheses are as follows: (1) the signal intensity (SI) ratio of the draining vein calculated by silent MRA is correlated with the arteriovenous transit time measured by DSA, and it can be used as a useful indicator of high-flow A–V shunt of BAVM, and (2) the characteristics of draining veins with the maximum rSI on silent MRA are associated with hemorrhage presentation.

## Material and methods

### Patients

The study protocol was approved by the Institutional Review Board of our institution. Written informed consent was obtained from all participants or their guardians at admission.

This retrospective study included 53 consecutive patients (mean age 33 years ± 16; 28 men) diagnosed with BAVM between May 2020 and February 2021 in a single tertiary referral institution. All patients underwent both silent MRA and DSA. Patients with MRA and DSA performed more than 4 weeks apart and patients with suboptimal imaging quality were excluded. Patients with subtentorial BAVM were not included in this study.

In patients without treatment, the initial presentation was divided into hemorrhagic or non-hemorrhagic by signs of bleeding on MRI consistent with hemorrhage history.

### Angiography

According to the standard protocol, DSA was performed for all the patients by the biplane system (Philips Medical Systems; Integris V5000). During the angiography, selective injection was performed in the vertebral arteries, internal carotid, and external carotid by using a 5-F catheter through a transfemoral route. Frontal and lateral views with a frame rate of four per second were displayed, and we injected a 5–7-mL bolus of the contrast medium iopromide (Ultravist; Schering) for each shot. In addition to the conventional DSA, a 3D rotational angiography was performed using 5 s, and thin slab-maximum intensity projection (MIP) and multiplanar reconstruction images were obtained.

### MRI protocol

3T MR imaging (SIGNA Premier; GE Healthcare) was performed within 4 weeks before or after DSA on all 53 patients. Patients did not receive microsurgery, endovascular treatment, or radiotherapy between MR imaging and DSA.

The conventional MR imaging protocol included axial T2WI (flip angle 8°; TR 4924 ms; TE 107.7 ms; section thickness 5.0 mm; NEX 1; bandwidth 62.50 kHz) and 3D sagittal T1 MPRAGE (flip angle 8°; TR 1000 ms; TE 2.7 ms; section thickness 1.0 mm; NEX 1; bandwidth 31.25 kHz).

The parameters of silent MRA were as follows: field of view (FOV), 20 × 20 cm; matrix, 150 × 150; flip angle, 5°; TR, 828 ms; TE, 0 ms; section thickness, 1.2 mm; NEX, 1; bandwidth, 25 kHz; and acquisition time, 6 min 59 s; and the labeling duration, 2034 ms. 3D radial sampling was applied during the readout scheme. A 48-channel head coil was used for all patients. To avoid the influence of the labeled position on the results, the lower edge of the FOV was located at the lower edge of the C2 vertebral body level in each patient.

### Image analysis

#### DSA image analysis

On DSA, the angioarchitecture and flow pattern (A–V transit time) of BAVM was evaluated by two interventional neurosurgeons (M.D. with 5 years of experience and T.H. with 10 years of experience) in a randomized order, with discrepancies resolved by consensus. Both of them were blinded to clinical information.

A–V transit time was estimated by determining the number of DSA frames between the first depiction of the nidus and the first visualization of a vein (high-flow A–V shunt—venous drainage seen in less than two frames after nidal visualization; non-high-flow A–V shunt—venous drainage seen in two or more frames after nidal visualization) [7]. Venous ectasia and stenosis were defined as at least 50% increase or reduction in the venous diameter in any drainage vein. Venous drainage was categorized as any deep venous drainage (exclusively deep venous drainage or superficial and deep drainage) or superficial venous drainage [18]. Flow-related aneurysm was defined as an aneurysm that lies on a pathway supplying blood flow to the BAVM [19]. The BAVM were further classified based on the Spetzler–Martin classifications by using DSA combined with conventional MRI.

#### Silent MRA analysis

Two neuroradiologists (C.W. and Z.Z., with 12 and 15 years of experience, respectively), blinded to the results of DSA and clinical data, independently reviewed all the conventional MR images and silent MRA images in a randomized order.

Measurements of the nidus were performed on the conventional MRI in three dimensions. The largest diameter among the three dimensions was recorded as the BAVM size. Location was classified as being in an eloquent or non-eloquent area.

**Fig. 1:**
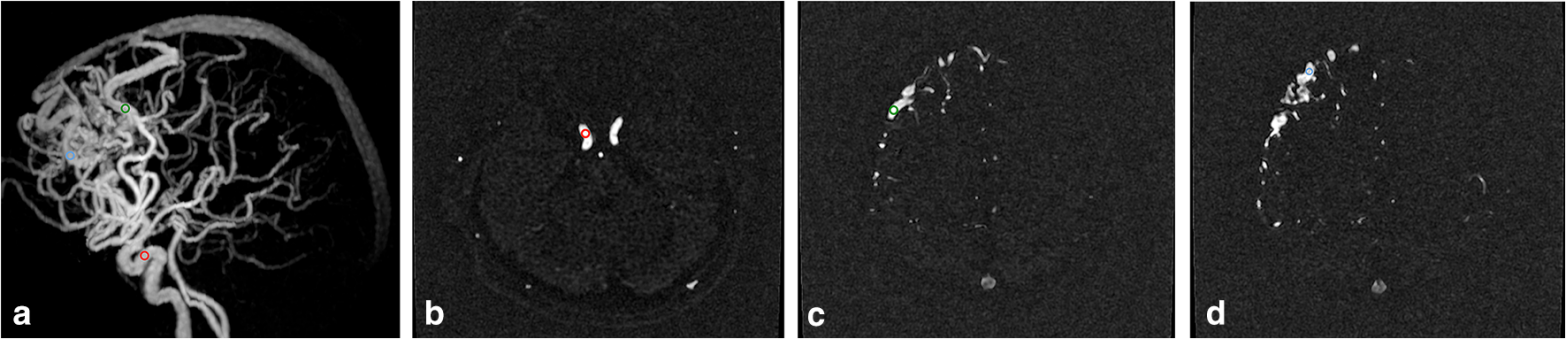
**a** ROI setting on Silent MRA. **b** ROIs were set on the cavernous ICA ipsilateral to the nidus (*red circle*) and (**c**-**d**) the proximal segment of drainage veins (green and blue circle) on axial source images. The ratio of signal intensity of each draining vein was calculated.

A region of interest (ROI) analysis was performed on the silent MRA source images on GE AW 4.7 Workstation. We used a circular shape for all ROIs. For draining veins (DV), the closest area to the nidus was first determined on MIP images. The ROI was put on the site on silent MRA source images and did not overlap with adjacent vessels (Fig. 1). To calculate the rSI, the cavernous ICA ipsilateral to the nidus was selected as the denominator for the rSI reference, which can easily be identified and measured on axial source images (refer to Fig. 1). For replication purposes, we performed similar analyses using the raw value of the SI of draining veins (see supplementary materials). Each ROI was set to be no larger than the target vessels, and the ROI size changed according to the vessel of interest. The ROI should exactly match the diameter of the vessel of interest. Each SI value was measured as the mean of 2 ROIs. Ratios of SI (rSI) were calculated as SI-DV / SI-ICA. The maximum ratio of SI (rSImax) was defined as the highest rSI of all draining veins in each patient. If the draining vein demonstrated on DSA cannot be visualized on silent MRA, the rSI was recorded as zero.

For each patient, the assessment included (1) rSI of each draining vein; (2) rSImax of all the draining veins; and (3) the diameter of the draining vein with rSImax.

Subsequent statistical analysis was based on the rSI values measured by the first neuroradiologist (C.W.). On DSA, the venous drainage patterns (venous ectasia, deep venous drainage, and single venous drainage) of the draining vein with rSImax were recorded.

### Statistical analysis

The data were analyzed by using IBM SPSS Statistics Version 22.0 (IBM). A *p* value < 0.05 was considered statistically significant. For the radiological and clinical data, patients with and without hemorrhage presentation before treatment were compared using descriptive statistics. Categorical variables were evaluated using Pearson’s chi-squared test and Fisher’s test, and continuous variables were evaluated using Student’s *t* test or the Mann–Whitney *U* test when comparing the two groups.

The intraclass correlation coefficient (ICC) of the different observers was calculated to assess the reliability of the rSI measurement. ICC was categorized as follows: values < 0.4 as poor, 0.40–0.59 as fair, 0.6–0.74 as good, and ≥ 0.75 as excellent [20].

The correlations of the rSI of each draining vein and the rSImax of each patient from silent MRA images with the corresponding A–V transit time from DSA were performed by Pearson correlation analysis. The same calculation was done for the correlations of rSImax with the corresponding diameter of the draining vein.

The area under the receiver operating characteristic curve (AUC-ROC) was obtained to explore the performance of high-flow A–V shunt determination by the rSI from silent MRA. A point with the maximum Youden index was set as the optimal cut-off. Sensitivity, specificity, positive predictive value, and negative predictive value were calculated.

## Results

### Patient demographics and BAVM characteristics

Of the 53 consecutive patients, seven were excluded for the following reasons: two patients had over 4 weeks interval between silent MRA and DSA, two patients had artifacts on silent MRA images, and three patients had a nidus in the posterior fossa. Among the 46 eligible participants, the mean age was 33.2 ± 16.9 years; range, 5–64 years; 25 men (Table 1). Twenty-six (56.5%) patients with BAVM were untreated, and 20 patients (43.5%) were previously treated by embolization and/or radiosurgery. Twenty-five patients (54.3%) had history of hemorrhage. Other symptoms (17 of 46; 37.0%) included seizure (9 of 46; 19.6%), headache (4 of 46; 8.7%), and dizziness (4 of 46; 8.7%). The BAVM was incidentally detected in 4 patients (8.7%). The mean interval between MR imaging and DSA was 5 days (range 1–15 days).

**Table 1:** Characteristics of the study population

Demographics	Total (n = 46)	Treated (n = 20)	Untreated		
			Hemorrhage (n = 12)	Non-hemorrhage (n = 14)	*p* value
Age at diagnosis (y)	33.2 ± 16.9	36.6 ± 16.7	27.2 ± 15.6	33.6 ± 18.0	0.69
Male	25 (54.3%)	11 (55%)	4 (33.3%)	10 (71.4%)	0.11
Radiological					
Venous drainage					
Single	21 (45.7%)	12 (60%)	4 (33.3%)	5 (35.7%)	1
Any deep	12 (26.1%)	4 (20%)	3 (25%)	5 (35.7%)	0.68
Venous ectasia	33 (71.7%)	12 (60%)	9 (75%)	12 (85.7%)	0.64
Deep location	3 (6.5%)	1 (5%)	3 (25%)	5 (35.7%)	0.68
Associated aneurysm	10 (21.7%)	1 (5%)	6 (50%)	3 (21.4%)	0.22
BAVM size (mm)	31.3 ± 17.5	27.5 ± 16.1	31.6 ± 19.0	37.4 ± 17.7	0.37
Spetzler–Martin grade					0.75
I–II	27 (58.7%)	13 (65%)	7 (58.3%)	7 (50%)	
III	13 (28.2%)	6 (30%)	3 (25%)	4 (28. 6%)	
IV–V	6 (13.0%)	1 (5%)	2 (16.7%)	3 (21.4%)	
High-flow A–V shunt	15 (32.6%)	5 (25%)	7 (58.3%)	3 (21.4%)	0.11

Values are numbers of patients; ages are mean ± standard deviation

*BAVM* brain arteriovenous malformation

Of all 46 patients, 46 niduses and 81 draining veins (54 draining veins in the untreated group and 27 in the treated group) were detected on DSA. High-flow A–V shunt was observed in 15 patients (10 of 26 in the untreated group and 5 of 20 in the treated group). Baseline characteristics are shown in Table 1. In patients without treatment, no significant difference was observed in any angioarchitecture features between the hemorrhagic and non-hemorrhagic groups.

### Association between the rSI on silent MRA and flow pattern

**Fig. 2:**
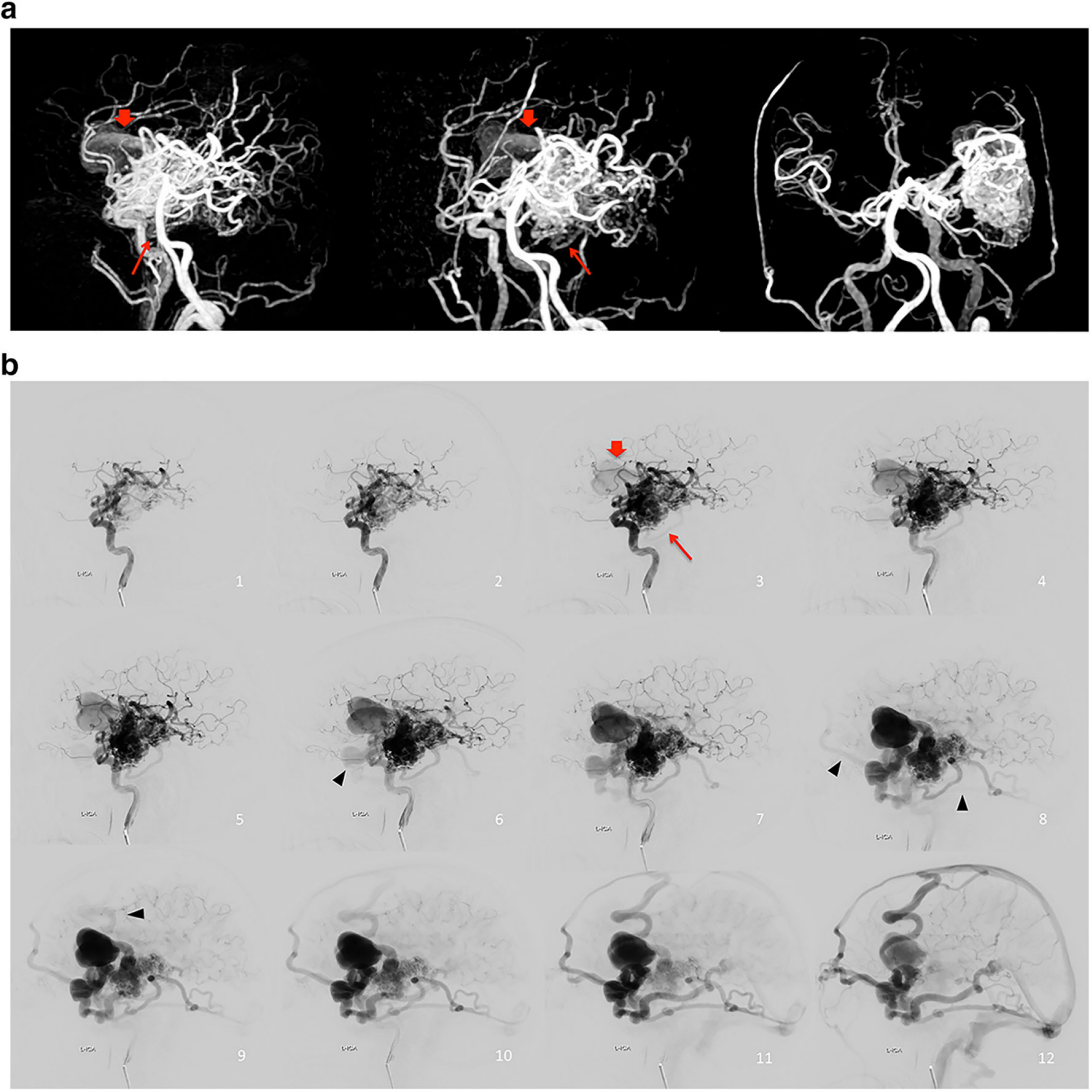
Images of left temporal BAVM in a 64-year-old man, with several asynchronous draining veins toward superior sagittal, sigmoid sinus and straight sinus. **a** 3D MIP of Silent MR angiography showed one dilated draining vein (red arrow) and a small draining vein (red long arrow). **b** Lateral view of left ICA angiography. These two veins were visualized two frames after the nidus depiction on DSA. Note that several draining veins (black arrowhead) observed more than four frames after the nidus depiction on DSA were not visible on Silent MRA.

All 46 niduses seen on DSA were detected on silent MRA. For all 81 draining veins, 73 (90.1%) (51 of 54 in the untreated group and 22 of 27 in the treated group) were detected on silent MRA. Five of these missing draining veins were toward the superior sagittal sinus and three were toward the sigmoid sinus. The corresponding A–V transit times on the DSA of these non-visualized draining veins were all more than four frames after nidal visualization (Fig. 2).

**Fig. 3:**
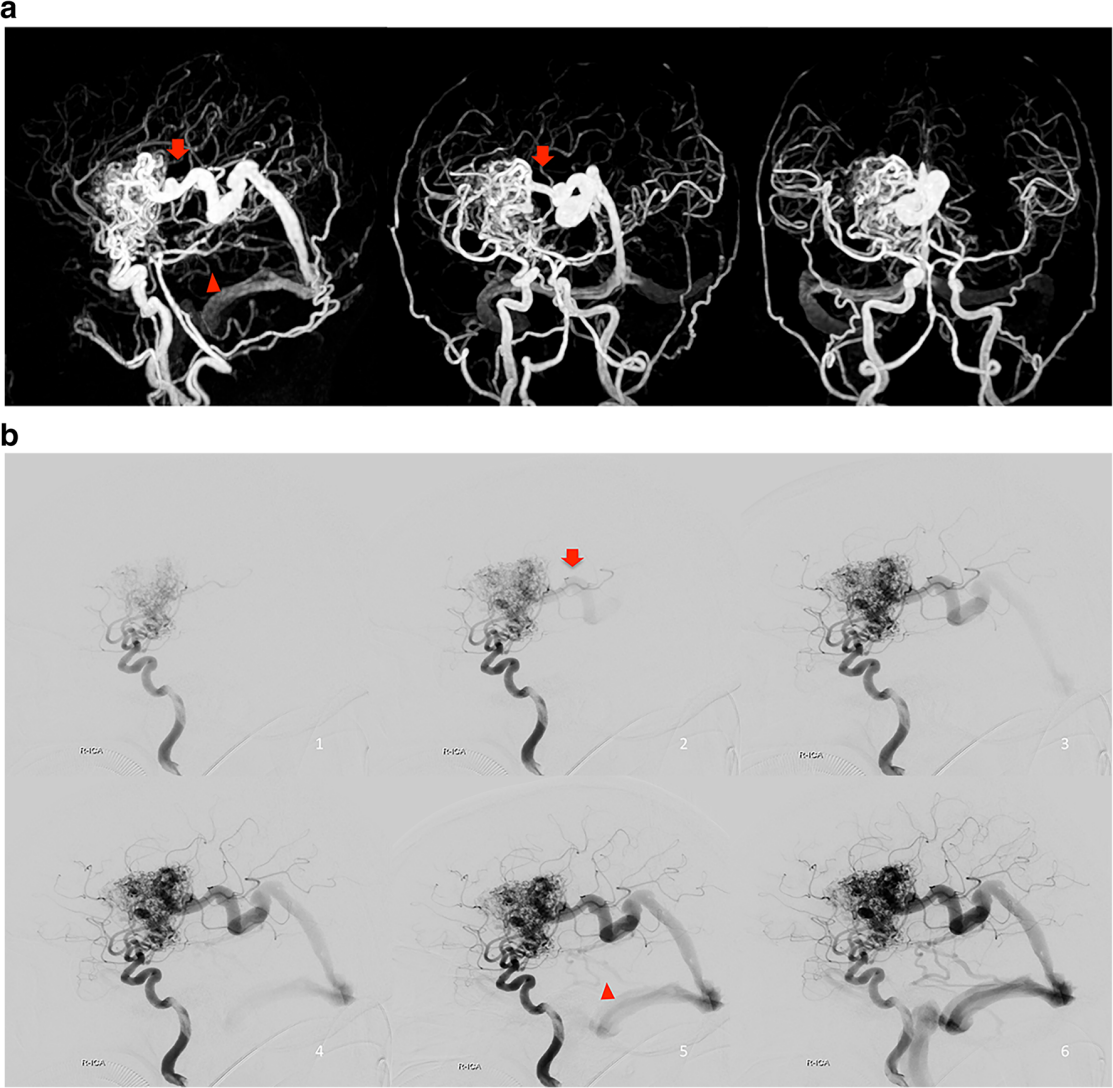
Images of right basal ganglia BAVM in a 33-year-old woman, with several asynchronous draining veins toward sigmoid sinus and straight sinus. **a** 3D MIP of Silent MR angiography showed one deep draining vein with higher signal intensity (red arrow) and several small draining veins with lower signal intensity (arrowhead). **b** Lateral view of right ICA angiography. The deep draining vein (red arrow) was visualized at the same time as one frame after the nidus depiction on DSA. The small draining veins with lower signal intensity on Silent MRA (arrowhead) were observed four frames after the nidus depiction on DSA.

For the ROI analysis, the ICC of the measurement of rSI was 0.89 (95% CI 0.77, 0.94). The rSImax of patients with high-flow A–V shunt was significantly higher than that of patients without high-flow A–V shunt (1.2 ± 0.17 vs 0.79 ± 0.37; *p* < .001). For draining veins with high-flow A–V shunt, rSI was significantly higher than that of draining veins without high-flow A–V shunt (1.2 ± 0.18 vs 0.74 ± 0.38; *p* < .001) (Fig. 3).

**Fig. 4:**
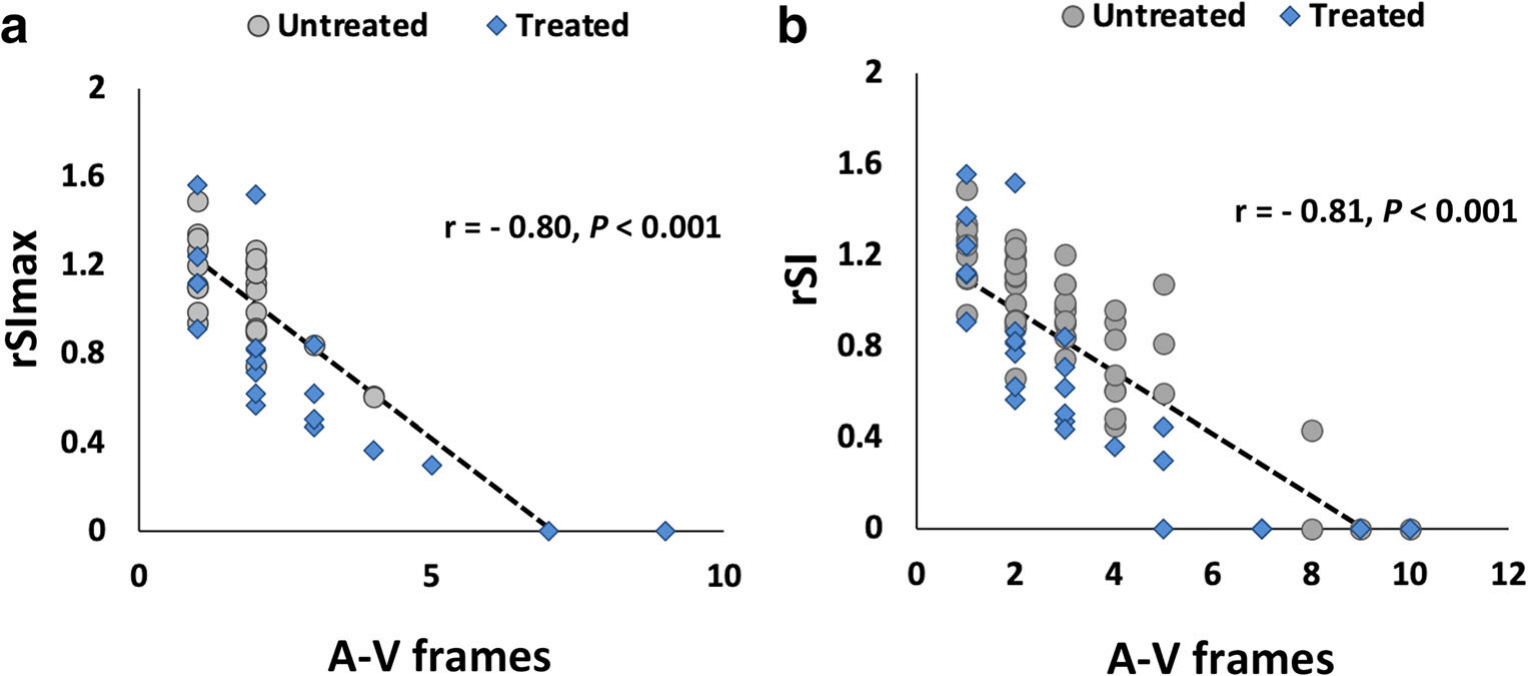
rSI versus arteriovenous transit time (**a**, rSI of each draining vein; **b**, rSImax)

The rSI of each draining vein and rSImax measured from silent MRA significantly correlated with the A–V transit time on DSA (*r =* −0.81, *p* < .001; *r =* −0.80, *p* < .001, respectively) (Fig. 4). This negative correlation was still significant in both patients treated (*r* = −0.83, *p* < 0.001 for rSI; *r* = −0.82, *p* < 0.001 for rSImax) and those untreated (*r* = −0.82, *p* < 0.001 for rSI; *r* = −0.65, *p* < 0.001 for rSImax). No significant correlation was observed between rSImax and the diameter of the corresponding draining vein (*r* = 0.18, *p* = 0.22).

Table 2 shows the diagnostic performance of rSI measured from silent MRA for the determination high-flow A–V shunt in treated and untreated patients using DSA as a reference. The area under the receiver operating characteristic curve (AUC-ROC) was 0.89 (95% CI 0.81, 0.96) for all patients. The rSI of 1.09 was set as the threshold for obtaining maximum sensitivity and specificity for high-flow A–V shunt determination. The sensitivity, specificity, positive predictive value (PPV), and negative predictive value (NPV) for high-flow A–V shunt were 82% (95% CI 55.8%, 95.3%), 82.8% (95% CI 70.9%, 90.7%), 56% (95% CI 35.3%, 75.0%), and 94.6% (95% CI 84.2%, 98.6%), respectively.

**Table 2:** Diagnostic performance of rSI measured by silent MRA to identify high-flow A–V shunt using DSA as a reference test

	AUC	Cut-off value	Sensitivity	Specificity	PPV	NPV
Total (n = 46)	0.885 [0.811, 0.960]	1.09	0.824 [0.558, 0.953]	0.828 [0.709, 0.907]	0.56 [0.353, 0.750]	0.946 [0.842, 0.986]
Treated (n = 20)	0.960 [0.882, 1.00]	0.88	1 [0.517, 1]	0.952 [0.741, 0.998]	0.857 [0.420, 0.992]	1 [0.800, 100]
Untreated (n = 26)	0.850 [0.735, 0.965]	1.09	0.818 [0.478, 0.968]	0.767 [0.610, 0.877]	0.474 [0.252, 0.705]	0.943 [0.794, 0.990]

95% confidence intervals are in brackets

*DSA* digital subtraction angiography, *AUC* area under the receiver operating characteristic curve, *A–V shunt* arteriovenous shunt, *DSA* digital subtraction angiography, *MRA* MR angiography, *NPV* negative predictive value, *PPV* positive predictive value, *rSI* ratio of signal intensity

### Association between the rSI on silent MRA and hemorrhage presentation

**Fig. 5:**
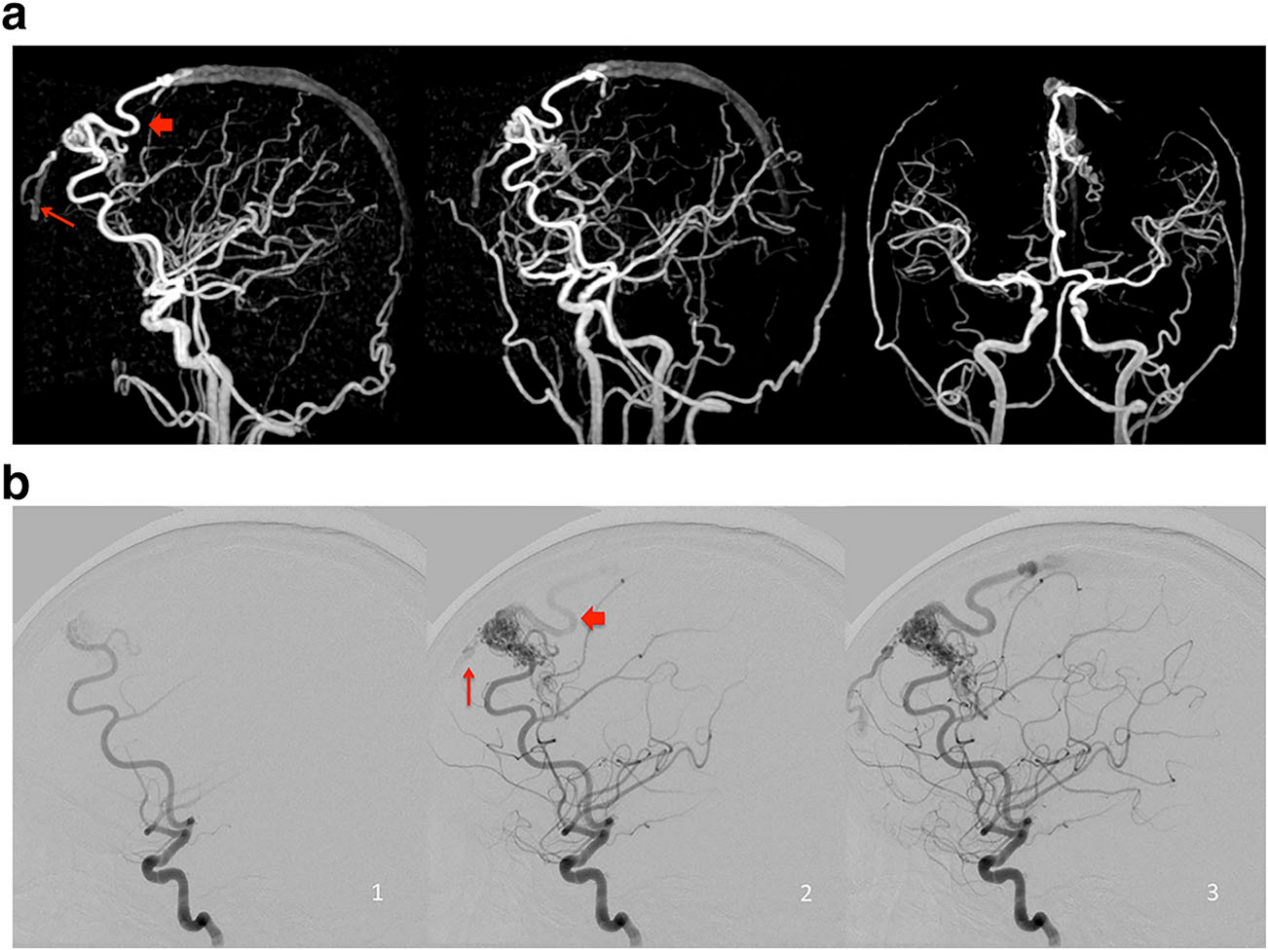
Images of left frontal BAVM in a 28-year-old woman with hemorrhage presentation. **a** 3D MIP of Silent MR angiography showed two draining veins with no ectasia (red arrow and long arrow) toward superior sagittal sinus. **b** Lateral view of left ICA angiography. These draining veins (red arrow and long arrow) were visualized one frame after depiction of the nidus on DSA, which were classified as high-flow A–V shunt.

In untreated patients, the difference of the presence of high-flow A–V shunt between the hemorrhage and non-hemorrhage groups was not significant (7/12 versus 3/14, *p* = 0.11) (Table 1), nor rSImax (1.1 ± 0.24 versus 1.1 ± 0.21, *p* = 0.70); however, the draining vein with a rSImax without venous ectasia was significantly observed in the hemorrhage group (*p* = 0.045) (Table 3, Fig. 5).

**Table 3:** Silent MRA relative signal intensity in patients without treatment associated with hemorrhage presentation

Demographics	Total (n = 26)	Hemorrhage (n = 12)	Non-hemorrhage (n = 14)	*p* value
rSImax	1.1 ± 0.22	1.1 ± 0.24	1.1 ± 0.21	0.70
DV with rSImax and no venous ectasia	11 (42.3%)	8 (66.7%)	3 (21.4%)	**0.045**
DV with rSImax and deep drainage	8 (30.8%)	5 (41.7%)	3 (21.4%)	0.40
DV with rSImax and single drainage	9 (34.6%)	4 (33.3%)	5 (35.7%)	1

Table entries are no. (%) or mean ± SD. The *p* value in bold type indicates statistical significance

*DV* draining vein, *MRA* MR angiography, *rSImax* maximum ratio of signal intensity

## Discussion

The assessment of arteriovenous (A–V) shunt was essential for risk stratification of untreated brain arteriovenous malformation (BAVM) and outcome evaluation in BAVM with endovascular or radiosurgical treatment. Therefore, an accurate, non-invasive imaging tool for A–V shunt analysis would facilitate the serial follow-up. The present study demonstrated that the ratio of signal intensity (rSI) measured by silent MR angiography (MRA) could be used to evaluate A–V transit time from digital subtraction angiography (DSA) in patients with BAVM. Results showed that there was a significant negative correlation between the rSI of draining veins from silent MRA and A–V transmit time from DSA (*r =* −0.81, *p* < .001). Furthermore, the rSI of the draining veins from silent MRA showed excellent diagnostic performance to identify the presence of high-flow A–V shunt with a reference to DSA (area under the receiver operating characteristic curve [AUC-ROC], 0.89; *p* < .001). The current study is, to the best of our knowledge, the first study to confirm that the rSI on silent MRA is a useful non-invasive tool for identifying high-flow A–V shunt of BAVM.

Seventy-three out of all 81 draining veins (90.1%) were detected on silent MRA in our study. Comparably, previous studies have also found that a small portion of draining veins of BAVM could not be visualized on silent MRA [11–14], yet few tried to explain this phenomenon. In the current study, the non-visualized draining veins were all observed more than 4 frames after nidal visualization on DSA, suggesting that those missing draining veins in silent MRA may be a consequence of slow blood flow. A similar observation was provided in a previous study by using silent MRA to evaluate cerebrovascular diseases [15], and the authors considered that the pattern of decreasing signal intensity distally within FOV on silent MRA was probably due to signal decays with the blood T1 weighted, which has been widely recognized in conventional arterial spin label imaging [21].

Because of T1 recovery, the signal decreases with the transit time from the labeling plane. Draining veins with different transit times experience different amounts of T1 recovery in silent MRA [16, 22]. In the current study, the rSI of the draining vein on silent MRA was significantly correlated with A–V transit time on DSA. This result interpreted that a shorter A–V transit time generally resulted in less T1 recovery and higher signal intensities of draining veins. Supportive evidence has been provided by a previous study using the SI on ASL to assess the degree of arteriovenous shunting on DSA [23]. Similar to our findings, they discovered that the BAVM signal intensity on ASL correlated well with the degree of early vein opacification on DSA, which corresponded to the degree of A–V shunting. However, the study was based on the ASL map that suffers from the intrinsic low spatial resolution and could not provide the anatomical detail of BAVM vasculature. In our study, the location of ROIs over both draining veins and arteries was easy to be identified on silent MRA, which made the measurement more feasible and reliable. In addition, we selected our ROIs over each draining vein at the point closest to the nidus to reduce the confounding contributions from physiological drainage [24, 25].

The distance from the labeling plane also possibly affects the signal intensity of the draining vein. To reduce the influence of distance on the results and ensure the reliability of measurement, we applied highly standardized localization strategy and used the cavernous ICA as the denominator for the rSI reference. Furthermore, we performed similar analyses using the raw value of the SI of draining veins (see supplementary materials), without considering the influence of distance. The results were still consistent with those using rSI, which suggested that the distance from the labeling plane did not play a major role in determining the signal intensity in our study.

In the present study, the rSI of the draining veins from silent MRA showed excellent diagnostic performance to identify the presence of high-flow A–V shunt. The excellent negative predictive value and lower positive predictive value of rSI suggest that this approach may be of use in ruling out high-flow A–V shunt in patients with BAVM in follow-up examination.

In the current study, the difference of the presence of high-flow A–V shunt and the maximum rSI between hemorrhage and non-hemorrhage groups was not significant. This result is consistent with a few previous studies. Shakur et al [26] and Lin et al [25] both discovered that the transnidal time measured from QDSA was not associated with hemorrhage presentation. Furthermore, we evaluated the characteristics of draining veins with the maximum rSI and found that no venous ectasia in conjunction with the maximum rSI was significantly more observed in the hemorrhage group. Similar results were reported in a previous study on subsequent hemorrhage in children with untreated BAVM [17] which found that the combination of fast A–V shunt and no venous ectasia significantly increased future hemorrhage risk in children with BAVM. Venous ectasia might be a protective factor and reflects an adaptive mechanism whereby such dysmorphism supports either a greater efferent capacitance and/or more functional arterialization of the draining veins [27]. Further research with a larger sample size is required to prove this hypothesis.

Our study had some limitations. First, the labeling plane of silent MRA was located at the lower edge of C2 vertebral body level, which is relatively low and might reduced flow sensitivity. However, a previous study found that the perfusion effect occurred at a higher labeling plane, resulting in incomplete subtraction and affecting the accuracy of signal intensity measurement [16]. In addition, the sample size of hemorrhage analysis was relatively small. This is a preliminary study with a limited sample size and selected patients; it should be very cautious to interpret the present findings in BAVM with variable location and hemodynamic features. However, the present findings provide an evidence for the feasibility of silent MRA for the evaluation of BAVM hemodynamics in further studies.

In conclusion, the SI ratio of draining veins measured by silent MRA was correlated with arteriovenous transit time of BAVM on DSA. The rSI on silent MRA could be used as a potential biomarker of high-flow A–V shunt of BAVM. Future studies could explore silent MRA in the rupture risk assessment and outcome evaluation after interventions for BAVM, such as target embolization and radiotherapy.

## Supplementary Information

ESM 1 (DOCX 22 kb)

## Abbreviations

ASL: Arterial spin labeling
AUC-ROC: Area under the receiver operating characteristic curve
A–V shunt: Arteriovenous shunt
BAVM: Brain arteriovenous malformation
CI: Confidence interval
DSA: Digital subtraction angiography
DV: Draining vein
FOV: Field of view
ICA: Internal carotid artery
ICC: Intraclass correlation coefficients
MIP: Maximum intensity projection
MRA: MR angiography
NPV: Negative predictive value
PPV: Positive predictive value
ROI: Region of interest
rSI: Ratio of signal intensity
rSImax: Maximum ratio of signal intensity
TOF: Time-of-flight
ZTE: Zero echo time

## Declarations

### Guarantor

The scientific guarantor of this publication is Jie Lu.

### Statistics and biometry

One of the authors (Chengbei Hou, PhD) has significant statistical expertise.

### Methodology

• retrospective

• diagnostic or prognostic study

• performed at one institution
