# Supplementary material for: Signal intensity ratio of draining vein on silent MR angiography as an indicator of high-flow arteriovenous shunt in brain arteriovenous malformation
Source: Eur Radiol. 2021 Jul 15;31(12):9252–61. doi: 10.1007/s00330-021-08170-8 (PMC8589750; doi:10.1007/s00330-021-08170-8)
Supplement: Supplementary file 1 — (DOCX 22 kb) [file 330_2021_8170_MOESM1_ESM.docx]

Supplemental Material

For replication purpose, we performed similar analyses using the raw value of SI of draining veins. We used a circular shape for all ROIs. The ROI was chosen at their closest area to the nidus and did not overlap with adjacent vessels

**Association between the SI on Silent MRA and flow pattern**

SImax of patients with high flow A-V shunt was significantly higher than that of patients without high flow A-V shunt (661 ± 198 vs 451 ± 240; *P* = 0.005). For draining veins with high flow A-V shunt, SI was significantly higher than that of draining veins without high flow A-V shunt (709 ± 97 vs 458 ± 230; *P* < .001).

The SI of each draining vein and SImax measured from Silent MRA significantly correlated with A-V transit time on DSA (r = - 0.80, *P* < .001; r = - 0.62, *P* < .001, respectively). This negative correlation was still significant in both patients treated (r = - 0.86, *P* < 0.001 for SI; r = - 0.56, *P* < 0.001 for rSImax) and those untreated (r = - 0.78, *P* < 0.001 for SI; r = - 0.52, *P* < 0.001 for rSImax). No significant correlation was observed between SImax and the diameter of the corresponding draining vein (r = 0.075, *P* = 0.62).

Table 1 showed the diagnostic performance of SI measured from Silent MRA for determination high flow A-V shunt in treated and untreated patients using DSA as a reference. The area under the receiver operating characteristic curve (AUV-ROC) was 0.87 (95% CI: 0.78, 0.96) for all patients. The SI of 634 was set as the threshold for obtaining maximum sensitivity and specificity for high flow A-V shunt determination. The sensitivity, specificity, positive predictive value (PPV) and negative predictive value (NPV) for high flow A-V shunt were 88% (95% CI: 62%, 98%), 80% (95% CI: 67%, 88%), 54% (95% CI: 32%, 72%) and 96% (95% CI: 86%, 99%), respectively.

**Table 2: Diagnostic Performance of rSI Measured by Silent MRA to Identify High flow A-V Shunt Using DSA as a Reference Test**

|  | AUC | Cut-off value | Sensitivity (%) | Specificity (%) | PPV(%) | | NPV(%) |
| --- | --- | --- | --- | --- | --- | --- | --- |
| Total (n=46) | 0.87 [78, 96] | 634 | 88 (15/17) [62, 98] | 80 (51/64) [67, 88] | 54 (15/28) [32, 72] | | 96 (51/53) [86, 99] |
| Treated (n=20) | 0.91 [78, 100] | 605 | 83 (5/6) [36, 99] | 86 (18/21) [62, 96] | | 63 (5/8) [26, 90] | 95 (18/19) [72, 100] |
| Untreated (n=26) | 0.87 [77, 97] | 669 | 82 (9/11) [48, 97] | 84 (36/43) [69, 93] | | 56 (9/16) [30, 79] | 95 (36/38) [81, 99] |

Note.—Numerator over denominator is in parentheses, and 95% confidence intervals are in brackets. DSA = digital subtraction angiography. AUC = Area under the receiver operating characteristic curve, A-V shunt = arteriovenous shunt, DSA = digital subtraction angiography, MRA = MR angiography, NPV = negative predictive value, PPV = positive predictive value, SI = signal intensity.

**Association between the SI on Silent MRA and hemorrhage presentation**

In untreated patients, the difference of the presence of high flow A-V shunt between hemorrhage and non-hemorrhage groups was not significant (7/12 versus 3/14, *P* = 0.11) (Table 1), nor SImax (635 ± 137 versus 647 ± 127, *P* = 0.84); however, draining vein with SImax without venous ectasia was significantly observed in the hemorrhage group (*P* = 0.045).

**Table 3. Silent MRA signal intensity in patients without treatment associated with hemorrhage presentation**

| Demographics | Total  (n=26) | Hemorrhage  (n=12) | Non-hemorrhage  (n=14) | *P* value |
| --- | --- | --- | --- | --- |
| SImax | 1.1 ± 0.22 | 1.1 ± 0.24 | 1.1 ± 0.21 | 0.70 |
| DV with SImax and no venous ectasia | 11/26 (42) | 8/12 (67) | 3/14 (21) | **0.045** |
| DV with SImax and deep drainage | 8/26 (31) | 5/12 (42) | 3/14 (21) | 0.40 |
| DV with SImax and single drainage | 9/26 (35) | 4/12 (33) | 5/14 (36) | 1 |

Note.—Unless otherwise noted, data in parentheses are percentages, values are numbers of patients; data in parentheses are percentages. SImax are mean ± standard deviation. DV = draining vein, MRA = MR angiography, SImax = maximum signal intensity.
